# GPs’ perspectives regarding their sedentary behaviour and physical activity: a qualitative interview study

**DOI:** 10.3399/BJGPO.2022.0008

**Published:** 2022-07-13

**Authors:** Richard S Mayne, Nigel D Hart, Mark A. Tully, Jason J Wilson, Neil Heron

**Affiliations:** 1 School of Medicine, Dentistry and Biomedical Sciences, Queen’s University Belfast, Belfast, UK; 2 School of Medicine, Ulster University, Newtownabbey, UK; 3 Sport and Exercise Sciences Research Institute, School of Sport, Ulster University, Newtownabbey, UK; 4 School of Medicine, David Weatherall Building, Keele University, UK

**Keywords:** sedentary behaviour, exercise, general practitioners, GP, primary health care, qualitative research methodology

## Abstract

**Background:**

General practice is a highly sedentary occupation, with many GPs spending more than 10.5 hours sitting each workday. This excessive sedentary behaviour and lack of physical activity (PA) is potentially detrimental to the health of GPs, as well as their ability to counsel patients regarding sedentary behaviour and PA. There is a lack of prior research examining the perspectives of GPs regarding their sedentary behaviour and PA.

**Aim:**

To explore GPs’ perspectives regarding their sedentary behaviour and PA.

**Design & setting:**

A qualitative interview study of GPs in Northern Ireland.

**Method:**

Semi-structured interviews were conducted with a purposive sample of 13 GPs who had previously taken part in a study to objectively measure their levels of sedentary behaviour and PA. Interview transcripts were analysed using deductive thematic analysis. The Theoretical Domains Framework (TDF) was used to facilitate identification of barriers and enablers affecting the ability of GPs to increase their PA.

**Results:**

Key themes were categorised within six theoretical domains (environmental context and resources, social professional role and identity, goals, social influences, knowledge, and intentions) with sub-themes within each domain.

**Conclusion:**

Most GPs are unhappy with their current levels of sedentary behaviour and PA, and are concerned with how this is affecting their health. Numerous barriers and facilitators were identified affecting the ability of GPs to increase their PA, including working environment, and personal and professional responsibilities, among others. Addressing these could improve the health of GPs and their ability to counsel patients regarding sedentary behaviour and PA.

## How this fits in

Excessive sedentary behaviour and insufficient PA is associated with many adverse health outcomes and increased all-cause mortality, yet little previous research has examined sedentary behaviour and PA among GPs. This study reveals that most GPs are unhappy with their current levels of sedentary behaviour and PA, and are concerned with how this is affecting their health. Numerous barriers and facilitators were identified affecting the ability of GPs to increase their PA. These factors should be addressed in order to improve the health of GPs and their ability to counsel patients regarding their PA.

## Introduction

Recent research conducted by the present authors has revealed that general practice is a highly sedentary occupation.^1^ Excessive sedentary behaviour (when someone is awake while sitting or lying in a state of low energy expenditure)^2^ and physical inactivity (an insufficient PA level to meet present PA recommendations)^3^ are associated with increased all-cause mortality and numerous adverse physical and mental health outcomes, in the form of a dose–response relationship, whereby increasing sedentary time corresponds with increasing mortality risk.^4–7^ The World Health Organization (WHO) 2020 guidelines on PA and sedentary behaviour advise individuals to minimise sedentary behaviour, and replace sedentary time with PA where possible.^8^ Furthermore, to reduce the detrimental effects of high levels of sedentary behaviour on health, adults should aim to do more than the recommended levels of moderate-to-vigorous PA.^8^

Many GPs typically spend more than 10.5 hours being sedentary during a typical workday.^1^ GPs with active workstations (such as height-adjustable sit–stand desks), however, typically spent 2.5 hours less time being sedentary during their workday.^1^ Given that GPs who are more physically active are more likely to recommend PA to their patients,^9–15^ and patients are more likely to make healthy lifestyle changes if they believe their doctor follows the same guidance themselves,^16–18^ encouraging GPs to sit less and move more could potentially lead to health benefits for both GPs and their patients. Like sedentary behaviour, cigarette smoking also has a dose–response relationship with mortality.^19^ Once the negative health effects of smoking were established, doctors were one of the first occupational groups to reduce their levels of smoking.^19–28^ This likely contributed to reduced levels of smoking among the general population, owing to the role of doctors in influencing the health behaviours of others.^16–26,27,27^ If GPs were to take action to reduce their sedentary behaviour and increase their PA, they could potentially play a similar role in encouraging wider society to follow suit.

The aim of this study was to conduct qualitative, semi-structured interviews with GPs, and use deductive thematic analysis to map their perspectives onto the TDF. The TDF was developed to investigate determinants of behaviour and inform the choice of potential strategies for behaviour change interventions.^29^ Specific focus was placed on identifying:

- Their knowledge of health outcomes related to sedentary behaviour and physical inactivity;
- Their own levels of sedentary behaviour and PA;
- The barriers and facilitators influencing their levels of sedentary behaviour and PA;
- The potential of workplace interventions to reduce sedentary behaviour and increase PA;
- How their own health behaviours affect their interactions with patients.

## Method

### Design

During autumn 2020, an online questionnaire, which was based on the International Sedentary Assessment Tool (ISAT),^30^ was distributed to all GPs and general practice specialty trainees (GPSTs) throughout Northern Ireland using email and social media, as described in a previous article.^1^ Participants were recruited voluntarily, with no obligations or rewards for taking part. Twenty questionnaire participants were recruited to a subsequent accelerometer and interview sub-study, with purposive selection to ensure maximal variation based on age, sex, work pattern or environment, access to an active workstation, and self-reported sedentary time. These participants were supplied with an Axivity (Newcastle-upon-Tyne) AX3 accelerometer (validated for the detection of sitting, lying, standing, and light, moderate and vigorous PA)^31^ to wear continuously on the middle of the thigh over a 7-day period, while completing a contemporaneous sleep/work log. They were subsequently approached to arrange an interview, during which they were advised that they would be informed of their average workday and non-workday sedentary time, standing time, light PA, moderate-to-vigorous PA, and step count, and asked questions relating to sedentary behaviour and PA.

### Data collection

One of the authors (RSM), a male academic GPST with previous experience in qualitative research, who had recruited the participants to the previous questionnaire and accelerometer studies, conducted in-depth semi-structured interviews over webcam (Microsoft Teams) during May and June 2021. The interview guide (Supplementary file 1) had previously been piloted and minor revisions were made to the questions after the first two interviews were conducted. The interview schedule addressed topics such as the following: how participants felt about their own levels of sedentary behaviour and PA inside and outside of work; awareness of health risks related to sedentary behaviour and PA; and possible interventions to reduce workday sedentary behaviour and increase PA. All participants had previously provided informed consent for the interviews, which lasted between 21 and 32 minutes in duration.

### Analysis

Interviews were recorded digitally and transcribed verbatim by the interviewer (RSM). Audio-recordings and transcriptions were stored securely using password protection, and were only available and accessible to the research team. Transcripts were coded within NVivo (version 12), using deductive thematic analysis^32^ to map viewpoints relating to GP sedentary behaviour and PA behaviour change onto relevant domains from the TDF.^33,34^ This study used the second version of the TDF, which was created after validation of the original TDF, and comprises 14 domains covering 84 theoretical constructs including social, environmental, cognitive, and affective components.^34^ Subsequent inductive analysis was undertaken to create explanatory sub-themes within the previously identified domains within the TDF.^35^ Independent analysis of a random sample of three interviews was initially undertaken by a second researcher (NH), who is a dual-qualified clinical academic GP and consultant in sport and exercise medicine. Differences in coding were discussed before a consensus was reached to ensure appropriateness of domain mapping and creation of sub-themes. The study was conducted in accordance with the consolidated criteria for reporting qualitative research (COREQ) checklist.^36^

## Results

**Figure 1.**
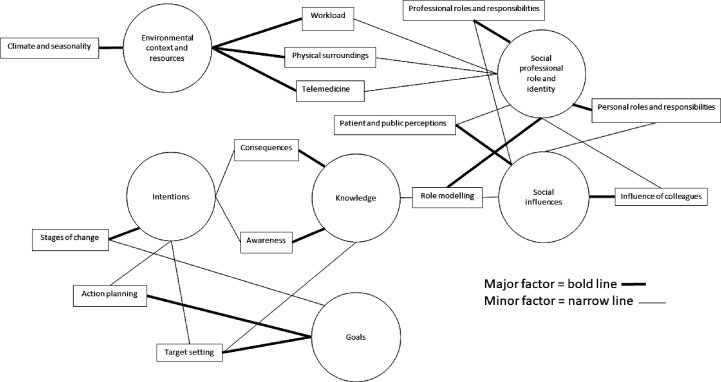
Explanatory map of key domains from the Theorectical Domains Framework and sub-themes influencing GP sedentary behaviour and physical activity

Ten GPs and three GPSTs participated in the interviews, comprising nine females and four males. Saturation of the main themes was reached after the 12th interview, as no new emerging themes were identified; however, a further interview was conducted to maximise participant diversity. Participants had between 1 year and 27 years of experience working in general practice. Three participants had experience of using active workstations in general practice, and 10 did not. Participants had recorded between 7.85 and 12.47 hours of average workday sedentary time during the preceding accelerometer sub-study. Key areas emerging from the interviews were categorised within six theoretical domains, with sub-themes within each. A map was used to identify the emerging relationships between domains and sub-themes, which identified strengths (major or minor factor) of the respective relationships and where they overlapped and interacted (Figure 1).^32^

### 1) TDF domain: Environmental context and resources

This is defined *'as any circumstance of a person’s situation or environment that discourages or encourages the development of skills and abilities, independence, social competence, and adaptive behaviour'*.^34^ Table 1 shows relevant participant quotations within this domain.

**Table 1.** Participants’ quotations for 'environmental context and resources' TDF domain

Sub-theme	Quote
Workload	*'It’s probably workload, obviously. The practice closes one to two, so really there shouldn’t be anybody turning up. Like, there’s no need for a GP to be in the building for that time. But there’s always paperwork, blood results, overdue phone calls that need done…*' (GP102, female)*'I often intend to go for a walk for 20 minutes at lunchtime, but sometimes the workload is so heavy that you think, well, if I go for a walk for 20 minutes at lunchtime, I’m going to be staying 20 minutes later at work … Some GPs, and I suppose I’m tempted now, bring a flask and have a cup of tea at their desk rather than having to walk down to the kitchen and waste five minutes doing that, you know, it’s just, it’s just the time pressure, isn’t it?*' (GP111, male)*'If I wasn’t in the room, which is the furthest from the waiting room, I might consider it … I think the human touch is very good, but yeah, by the time I walk to the waiting room and walk back again it takes too long.'* (GP 110, male)
Climate and seasonality	*'I guess during winter time I find it very difficult to get up in the morning in the ice and the cold to get out for any sort of exercise.'* (GP109, female)*'If it’s wet and dreary, I probably prefer just to push on with some work rather than head out to walk and get soaked.*' (GP102, female)
Physical surroundings	*'It’s a really tiny practice, so we don’t have long corridors or stairs or anything like that.'* (GP108, female)*'When I was in my previous practice, I sometimes would have gone out for even a 15–20-minute walk. There was a small walkway by the practice I was working at. So sometimes would have done that just to clear the head but there’s not really the same opportunities to do that in my current workplace … It wouldn’t be the most appealing place to go for a walk whereas the country practice I was in before had a dedicated eco-garden and walkway, which was quite pleasant.*' (GP 113, male)*'Just the way my room is set up, we have shelves above our desks so … I thought there was going to be a lot of hassle, trying to practically install it.'* (GP 102, female)
Telemedicine	*'Prior to the pandemic, prior to doing telephone triage, I would have walked, I’m not going to pretend it was much, but it would have been a bit more because we didn’t, you know, we would have gone out and called patients. At least you got up. Whereas on the telephone you just literally sit in your chair and that’s you, you know, for three hours solid and then you see the three or four patients you have booked in, so there’s very little opportunity for activity at all in the workplace. But I don’t know how you change that.'* (GP112, female)*'At the moment telephone triage is making us all slower and fatter.'* (GP110, male)*'If you wanted to make changes you could use* [telemedicine] *as a positive. Because obviously if you do a telephone consultation, you could make a handstand or lie in your bed or sit or stand, whatever, you know, it wouldn’t make much of a difference. It would be different, of course, if you have a video consultation. Then you also need to have a comfortable relaxed body language, which is probably better to achieve when you’re sitting. But if it’s just a plain telephone consultation, you could do it standing.'* (GP104, male)

TDF = Theorectical Domains Framework

#### 1.1) Sub-theme: Workload

All participants identified workload as a key barrier to reducing sedentary behaviour and increasing PA. The working day is extremely busy, with limited time for breaks, impacting the time available to leave the consulting room. This was true for both GPSTs and GPs, as well as those working in urban, suburban, and rural environments. One participant identified how the time taken for non-patient-facing tasks often spilled over into the lunchbreak, which limited the opportunity for PA breaks. Another participant identified how GPs can be reluctant to move away from their desk owing to time pressures. Time taken to walk to the waiting room to greet patients was also felt to be a deterrent for some participants.

#### 1.2) Sub-theme: Climate and seasonality

As with the general population, weather and seasonal factors affected willingness of participants to engage in PA. This was especially relevant given the relatively wet weather in Northern Ireland.^37^ One participant cited being deterred by cold conditions, while another participant identified wet weather as a barrier to PA during the lunchbreak, instead opting to continue with work-related tasks.

#### 1.3) Sub-theme: Physical surroundings

The built and natural environment in and around the workplace influenced PA. One participant, a GP partner, cited a small building as limiting the opportunity for movement. Another participant, a GPST, identified the area surrounding their workplace as being either a positive or a negative influence on their desire to walk when they had the opportunity. Another participant had considered installing a height-adjustable sit–stand desk, but was deterred by the layout of their room.

Physical surroundings are especially relevant for GP partners who often work in the same building for many years. They can potentially have a degree of control over their physical environment, such as installing a sit–stand desk in their consulting room, which GPSTs and sessional GPs may be unable to do. GPSTs and sessional GPs, however, typically work in a range of different locations, meaning they can encounter more variety in the built and natural environments where they are working.

#### 1.4) Sub-theme: Telemedicine

Remote consulting was identified by many participants to be a cause of excessive sedentary time. This is particularly relevant now that GPs provide a high volume of remote consultations, traditionally performed while sitting down, which were initially a way of providing safe patient care at the outset of the COVID-19 pandemic, but will likely remain a permanent part of general practice moving forward.^38,39^ However, some recognised the possibility of telemedicine to allow an increase in PA.

### 2) TDF domain: Social professional role and identity

This encompasses *'a coherent set of behaviours and displayed personal qualities of an individual in a social or work setting'*.^34^ Table 2 shows relevant participant quotations within this domain.

**Table 2.** Participants’ quotations for 'social professional role and identity' TDF domain

Sub-theme	Quote
Professional roles and responsibilities	*'It’s a whole cultural thing, isn’t it? But I, we, can start. We can start somewhere as a practice ... We shouldn’t need to wait from on high to be told what everyone’s going to do. We should make a start, you know. But yeah, I guess it’s just that we get so bogged down with the day-to-day work, don’t we? And we just, we neglect ourselves.'* (GP106, female)*'There’s no sort of* [physical] *activity throughout the day ... It’s just constant. I’m sitting on my bum from half eight until half one, eat my lunch at my desk and sit again till six. There’s literally no activity in my day at work.'* (GP109, female)
Personal roles and responsibilities	*'Young kids don’t make it easy. There’s always other things to pull away your time.'* (GP102, female)*'*[At the end of the working day] *I would rather get home and see the kids and plan to do some exercise after they’re in bed. But you feel less inclined at eight o’clock at night to do that.*' (GP111, male)*'If I didn’t walk the dog every morning … my step count would be absolutely dire. And it’s the one thing that does sort of keep me more active, I think.'* (GP108, female)
Role modelling	*'Those who come to GPs are very often those who are less fortunate in life. So, I think if you are too high up there, it’ll be very difficult for somebody who’s inactive and overweight to admit to that if every GP they go to is the embodiment of physical health. Of course, it has to be a bit of a balance. If you come to your GP and he’s still smoking and has a glass of whiskey on his desk, that’s probably not so good an idea.'* (GP104, male)*'At the end of the day, you’re trying to promote. And how do you promote a healthy lifestyle? By actually trying to lead a similar healthy lifestyle.'* (GP 118, female)

TDF = Theorectical Domains Framework

#### 2.1) Sub-theme: Professional roles and responsibilities

Some participants’ beliefs about their professional roles influenced their perceptions regarding their abilities to make changes to their sedentary behaviour and PA, especially within a wider workplace context.

#### 2.2) Sub-theme: Personal roles and responsibilities

Family commitments, particularly among participants with young children, were identified as limiting the time available for PA. Some personal roles were identified as having a positive influence on PA such as having a pet dog. This aligns with previous research, which demonstrated that dog ownership is associated with increased PA.^40–44^ However, just two participants mentioned that they owned a dog. This may be owing to high workload and long core working hours of 08:00–18:30 each weekday,^45^ which may make GPs feel less capable of owning a dog than people working in other occupations.

#### 2.3) Sub-theme: Role modelling

All participants felt that their own lifestyle choices affected their ability to effectively counsel patients regarding health behaviours; however, the importance of this varied between participants.

### 3) TDF domain: Goals

These are the *'mental representations of outcomes or end states that an individual wants to achieve'*.^34^ Table 3 shows relevant participant quotations within this domain.

**Table 3.** Participants’ quotations for 'goals' TDF domain

Sub-theme	Quote
Target setting	*'I'm trying, if possible, to get above this 10 000 steps a day, whatever good that may bring. And I'm so far on average, probably three times a week, I'm able to do that.'* (GP104, male)*'We had a practice step challenge to see who could get the most number of steps in a day. I did that for a while but I was thoroughly depressed because I had a very, very low* [number of steps].' (GP110, male)
Action planning	*'I think the biggest issue in modern life limiting exercise would be people saying that they’re too busy. But I mean, you know, clearly we are busy people. And if we can't afford three mornings a week … to get out and get a run, or whatever your exercise is … I think it’s just so important for your physical and mental health, and I think it shows. It shows to others that no matter how busy you are, you can fit something in or prioritise it. It’s about priorities.'* (GP109, female)*'If people are determined they will always find time to do things. But they have to find that time, they have to commit to it, and they have to sacrifice sitting in front of the TV.'* (GP118, female)

TDF = Theorectical Domains Framework

#### 3.1) Sub-theme: Target setting

Many participants monitored their levels of PA, such as with mobile-phone apps or wrist-worn activity trackers, and set targets for themselves, which were generally deemed to be helpful. However, one participant identified how having a goal for PA could be problematic if they felt unable to achieve it. Monitoring technology was equally prevalent among male, female, younger, and older participants, which reflects how most members of society are becoming increasingly technology literate over time.^46^

#### 3.2) Sub-theme: Action planning

Some participants deemed that prioritising PA was essential for physical and mental wellbeing; however, some views could potentially be seen as being judgemental of people deemed not to be meeting PA recommendations.

### 4) TDF domain: Social influences

This represents the *'interpersonal processes that can cause individuals to change their thoughts, feelings, or behaviours'*.^34^ Table 4 shows relevant participant quotations within this domain.

**Table 4.** Participants’ quotations for 'social influences' TDF domain

Sub-theme	Quote
Influence of colleagues	*'*[Getting out for lunchtime walks] *was quite nice actually. You know, it was nice surroundings as well, so that was great and just doing it together meant that there was like a peer drive to go.'* (GP119, female)*'I probably thought I’ll get mocked by my partners for having a standing desk ‘cause I’m always trying to come up with new ideas.'* (GP111, male)
Patient and public perceptions	*'The problem is also because you’re right in the middle of the community. If they see the doctor out taking a walk they’ll think, “Oh they’re not that busy,” you know, “they’ve got time to be walking down the beach every day,” you know. I mean, “My goodness, what are they being paid for?” So, I don’t know. There’s that sort of balance sometimes. You know, if I were to be out, I kind of almost don’t want to see any of the patients, but you know, it’s very unlikely that if you’re out in the middle of the day, you’re not going to bump into, you know, everyone you bump into is going to be your patient. And even though you might not recognise them, they certainly will recognise you.'* (GP108, female)*'I know that when the reception staff go in even to* [the supermarket] *to buy some lunch they’ve been, you know, like verbally abused by patients for being out when they can’t get appointments, you know, or when they can’t get through to the practice. So that has been in my mind, but where I’m working at the moment is a big enough place … so it’s a bit easier to be anonymous.'* (GP113, male)

TDF = Theorectical Domains Framework

#### 4.1) Sub-theme: Influence of colleagues

Colleagues provided both positive and negative influences on the likelihood of participants to engage in behaviours to reduce their sedentary behaviour and increase their PA. This was valid for both GP partners and GPSTs, despite the expectation that partners would feel more deeply imbedded and established in their practices, and thus potentially less likely to be influenced by others.

#### 4.2) Sub-theme: Patient and public perceptions

How participants felt they were viewed by patients and members of the public were important factors when they were deciding on the pros and cons of engaging in PA, particularly near where they worked. This overlapped with the role-modelling sub-theme, as described above. The balance of being a positive role model for PA among patients and colleagues, such as by getting out to exercise during the lunchbreak, clashed with the perception of needing to be seen to be working hard by the general public.

### 5) TDF domain: Knowledge

This is *'an awareness of the existence of something'*.^34^ Table 5 shows relevant participant quotations within this domain.

**Table 5.** Participants’ quotations for 'knowledge' TDF domain

Sub-theme	Quote
Consequences	*“Well, it’s the usual … weight gain, diabetes, heart, blood pressure … problems with your joints. Stress probably as well. Mental health. Yeah, everything.'* (GP106, female)*'Almost every condition is made worse by lack of physical activity … but obviously particularly cardiovascular risk and metabolic syndrome and diabetes. But then even things like mental health problems, depression, anxiety, and so on. Any chronic disease really is. You're increasing your risk by a lack of physical activity.'* (GP111, male)
Awareness	*'I'm always a bit cautious if you have an initiative which, em, tries to get people to do one thing or whatever, you know. I think it’s better if you are able to integrate things into your daily routine … For example, like your* [commute] *to work. Once you change that, you can either walk or cycle and if that becomes a habit then that’s for life, you know. Likewise, if at work if you establish that, “Right, when I finish seeing my patients and I look at my results, that’s when I stand up,” or, “I do that on a different computer where I have to stand,” I think then there’s a better chance that you maintain it. Rather than if you have an initiative which runs out at some stage.'* (GP104, male)*'And I know that there’s like parkrun for GP surgeries on Saturdays … but I don't think for me a parkrun on a Saturday is going to outdo the Monday to Friday concerns.'* (GP109, female)

TDF = Theorectical Domains Framework

#### 5.1) Sub-theme: Consequences

All participants had good understanding and awareness of health risks related to excessive sedentary behaviour and physical inactivity. This is likely a further source of internal conflict for many GPs, who are aware of the benefits of PA for their own health and wellbeing, but not always able to achieve the recommended levels of PA for various reasons described above.

#### 5.2) Sub-theme: Awareness

Awareness of interventions and initiatives to increase PA among general practice staff was mixed. Ten participants (76.9%) were aware of the parkrun practice initiative,^47^ but only one participant (7.7%) had heard of the Royal College of General Practitioners (RCGP) Active Practice Charter.^48^ Some participants were also sceptical about the effectiveness of these interventions and initiatives. This aligns with research showing that people are more likely to maintain positive health behaviours if they make them habitual, and that high levels of PA are required to offset the harms of excessive sedentary behaviour.^49,50^

### 6) TDF domain: Intentions

This is when someone makes a *'conscious decision to perform a behaviour or resolves to act in a certain way'*.^34^

#### 6.1) Sub-theme: Stages of change

All participants had different levels of motivation regarding their willingness to change their levels of sedentary behaviour and PA. Individuals typically cycle between stages of change at different time points depending on internal and external factors throughout their life course.^51^

The following partcipant was in the contemplation stage:

*'I probably feel like I'm not as active as I should be, probably. I think the NHS recommend 150 minutes, well I don’t know, 150 minutes per week of light-to-moderate exercise and then over 70 minutes of vigorous whereas I kind of feel like. I mean I just go for a few walks, but other than that don't do any other physical activities like running or cycling or sport at the moment. So I feel like I could definitely improve on that.'* (GP119, female)

The following participant was in the maintenance stage:

*'I just make sure … throughout the morning, I'll periodically take my scripts through. So I make a point, maybe after every four or five patients of just getting up, putting the headset off and walking away from my desk just to make sure that I stand up and maybe force myself to take a bathroom break every, at least every 10 patients as well.'* (GP113, male)

## Discussion

### Summary

Despite widespread awareness among GPs of the health risks associated with physical inactivity, and of the current UK PA guidelines, they identified many barriers that hindered their own abilities to reduce their sedentary behaviour and increase their PA. These included workload, personal and professional roles and responsibilities, and physical infrastructure. Several factors were seen as being enablers for some, and barriers for others. These included telemedicine, the local environment, social influences, patient and public perceptions, and climate and seasonality. GPs did recognise their potential to be positive role models in encouraging patients and colleagues to become more physically active. They were aware that for this advice to seem credible to patients, GPs should aim to be less sedentary and more physically active themselves. The idea of GPs being a positive role model for PA was tempered, however, by concerns of a negative public perception if GPs were seen to be taking breaks for PA during the working day. This is especially owing to present concerns around patient access and primary care workload. It, therefore, appears that many GPs do not currently feel able to adopt the role of being a positive PA role model to their patients, which represents a missed opportunity for healthy lifestyle promotion and the associated benefits this could entail.^52^

### Strengths and limitations

This study has provided detailed insights into the thoughts and opinions of GPs regarding their own sedentary behaviour and PA, with interviews conducted after participants had received detailed, personalised, objective feedback regarding their sedentary behaviour and PA throughout a 7-day period. Although many studies have previously focused sedentary behaviour and PA among patients and the general public, minimal previous research has examined this among GPs themselves. Although all participants were working in Northern Ireland at the time of the study, they had similar working conditions to their colleagues working within general practice across the rest of the UK.

This study only examined the perspectives of GPs and GPSTs; however, more diverse views could potentially be identified by including other professions, such as nurses, receptionists, and allied health professionals working in the general practice setting.

Using deductive analysis to map responses to domains within the TDF meant that some responses could potentially have been mapped to more than one domain. However, when this was the case, the domain with most perceived relevance was chosen, as shown in Figure 1.

### Comparison with existing literature

Findings from this study align with similar previous research among other population groups both inside and outside the field of medicine. Despite many GPs having good knowledge of PA guidelines, counselling patients about the benefits of PA is often a low priority compared with other health behaviour modifications.^53^ Therefore, although GPs could be in an optimal position to counsel their patients about the health benefits of PA, this is not currently being realised.^53^ In a cross-sectional survey of GPs in the Republic of Ireland, time pressures, lack of energy, lack of suitable exercise facilities and environment, and competing roles and responsibilities were all identified as barriers to them increasing their own PA.^54^ Similar findings were reported among doctors and nurses working in India, with participants additionally reporting that they prioritised the health of their patients over their own health.^55^ Desk-based workers in other professions also identified similar barriers to increasing their PA such as workload, workplace culture, social norms, and public perceptions.^56^ Cultural and social factors were also important among other population groups, such as university students^57^ and older adults,^58^ demonstrating that these are relevant considerations across the life course.

Numerous positive aspects relating to PA were identified in this study, including both physical and mental health benefits. This corresponds with previous research examining motivations for participation in PA among other desk-based workers^56^ and individuals across the lifespan.^59^ In terms of enablers that helped participants to be more physically active, a positive workplace culture, self-monitoring, goal-setting, and social support were all identified by nurses in Queensland, Australia,^60^ which aligns with the findings from this study. In general, therefore, this study demonstrates that GPs have similar barriers and facilitators to increasing their PA as most other population groups, with the additional awareness and insight into how their current high levels of sedentary behaviour and physical inactivity are negatively affecting their own health. However, despite this knowledge, it appears that for many GPs there are more barriers, which play a stronger role than enablers, to them reducing their sedentary behaviour and increasing their PA. Changing general practice workplace culture through behaviour change interventions requires a complex, multi-layered process,^61^ which goes above and beyond the current offerings relating to PA promotion; however, recent initiatives such as the RCGP Active Practice Charter can play a positive role.^48^

Increasing evidence is emerging of the positive effects of interventions to reduce sedentary behaviour through improving biomarkers of cardiometabolic risk.^62^ Several studies have shown the efficacy of multicomponent interventions in helping to reduce sedentary time and increase PA among desk-based workers.^63–65^ Co-production of 'sit less at work' interventions, taking pragmatic approaches to reduce barriers to PA, appear promising among smaller workplaces, and could potentially be replicated among general practices in future.^66,67^ Like other behaviour change interventions, for this to be successful in the general practice setting, careful consideration is needed of the many factors that contribute to change management at the individual and organisational level, to ensure that enablers, to GPs reducing their sedentary behaviour and increasing their PA, become stronger than the many barriers identified in this study.^67,68^

### Implications for research and practice

This study has demonstrated that GPs have good awareness of the negative health consequences of excessive sedentary behaviour and physical inactivity, and that most GPs are unhappy with their current levels of sedentary behaviour and PA. Numerous barriers and facilitators to GPs increasing their PA have been identified. Therefore, further research should assess the acceptability of co-produced multicomponent interventions aimed at encouraging general practice staff to be less sedentary and more physically active throughout the working day, as well as how this affects their interactions with patients and ability to counsel patients about PA.

## Data

As this is a qualitative study, the dataset is not publicly available to minimise likelihood of participants being identified.
